# Matrix Metalloproteinase 13 (MMP13) Is a Direct Target of Osteoblast-Specific Transcription Factor Osterix (Osx) in Osteoblasts

**DOI:** 10.1371/journal.pone.0050525

**Published:** 2012-11-21

**Authors:** Chi Zhang, Wanjin Tang, Yang Li

**Affiliations:** Bone Research Laboratory, Texas Scottish Rite Hospital for Children, University of Texas Southwestern Medical Center, Dallas, Texas, United States of America; National University of Singapore, Singapore

## Abstract

Osterix (Osx) is an osteoblast-specific transcription factor required for bone formation and osteoblast differentiation from mesenchymal stem cells. In *Osx*-null mice, no bone formation occurs. Matrix metalloproteinase 13 (MMP13) is a member of the matrix metalloproteinase family and plays an important role in endochondral ossification and bone remodeling. Transcriptional regulation of *MMP13* expression in osteoblasts is not well understood. Here, we provide several lines of evidence which show that MMP13 is a direct target of Osx in osteoblasts. Calvaria obtained from *Osx*-null embryos displayed dramatic reductions in MMP13 expression compared to wild-type calvaria. Stable overexpression of Osx stimulated MMP13 expression in C2C12 mesenchymal cells. Inhibition of Osx expression by siRNA led to downregulation of MMP13 expression. Mechanistic approaches using transient transfection assays showed that Osx directly activated a 1 kb fragment of the MMP13 promoter in a dose-dependent manner. To define the region of the MMP13 promoter that was responsive to Osx, a series of MMP13 promoter deletion mutants were examined and the minimal Osx-responsive region was refined to the proximal 80 bp of the MMP13 promoter. Additional point mutant analysis was used to identify one GC-rich region that was responsible for MMP13 promoter activation by Osx. Gel Shift Assay showed that Osx bound to *MMP13* promoter sequence directly. Chromatin immunoprecipitation assays demonstrated that endogenous Osx was associated with the native MMP13 promoter in primary osteoblasts *in vivo*. Taken together, these data strongly support a direct regulatory role for Osx in MMP13 gene expression in osteoblasts. They further provide new insight into potential mechanisms and pathways that Osx controls bone formation.

## Introduction

Bone formation is a highly regulated process involving the differentiation of mesenchymal stem cells to osteoblasts. It involves two processes: intramembranous and endochondral ossification. The majority of bones in the mammalian skeleton form by endochondral ossification which requires a cartilagenous template for osteoblast-directed mineral deposition. Fewer bones, particularly those in the skull, form by intramembranous ossification, a process in which mineralized bone forms directly from mesenchymal condensations without cartilage template. Osteoblast differentiation from mesenchymal stem cells occurs through a multi-step molecular pathway controlled by a complexity of transcription factors and signaling proteins, including Indian Hedgehog, Runx2, Osterix (Osx), and Wnt pathway proteins [1]. Indian Hedgehog is required for endochondral ossification and is needed for the activation of Runx2 [2]. Both endochondral and intramembranous ossification require Runx2 which is involved in the differentiation of mesenchymal cells into preosteoblasts [3]. As a downstream gene of Runx2, Osx is specifically expressed in osteoblasts and at low levels in prehypertrophic chondrocytes [4]. Osx is essential for the commitment of preosteoblastic cell differentiation into mature osteoblasts. Wnt signaling plays a role in osteoblast differentiation and proliferation, and is important for bone mass determination in the adult bone. Wnt pathway affects different stages of bone formation and bone metabolism [5].

Osx is an osteoblast-specific transcription factor required for bone formation and osteoblast differentiation. Osx knock-out mice completely lack mineralized bone while cartilagenous tissue is essentially normal [4]. Osx-null mouse embryos do not express osteoblast differentiation markers, such as osteocalcin (OC), alkaline phosphatase, and others. Osx is reported to inhibit the Wnt signaling pathway, providing a possible mechanism through which Osx inhibits osteoblast proliferation [6]. It is suggested that Osx coordinates both osteoblast differentiation and osteoblast proliferation during bone formation. The observation that Osx inhibits the Wnt pathway highlights the potential for novel feedback control mechanisms involved in bone formation.

**Figure 1:**
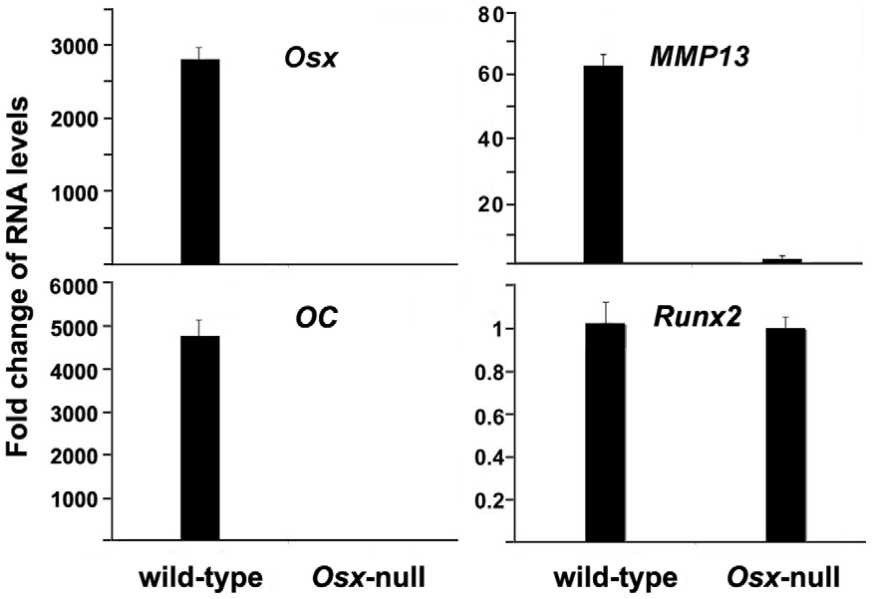
Osx ablation reduces MMP13 gene expression in vivo. Calvaria RNAs were isolated from E18.5 *Osx* wild-type and *Osx*-null embryos. RNA expression levels for Osx, osteocalcin (OC), Runx2 and MMP13 were analyzed by real-time RT-PCR. The level of each RNA from *Osx*-null calvaria was normalized to a value of 1. Values are presented as the mean ± S.D.

Matrix Metalloproteinase 13 (MMP13, also called collagenase-3) is a member of the large family of matrix metalloproteinases. The MMPs are a family of extracellular matrix-degrading enzymes that share several structural features in common including the presence of a conserved zinc-binding catalytic domain. These enzymes are synthesized as secreted transmembrane proenzymes and processed to the active form by removal of an amino-terminal propeptide. The MMPs are endopeptidases that regulate cell growth, migration, and extracellular matrix remodeling [7]. Of the MMPs, MMP13 has been considered to have an essential role in bone biology in view of its exclusive presence in the skeleton during development. MMP13 plays an important role in the degradation of components of the extracellular matrix, particularly the collagens. It degrades collagen type II efficiently but also collagens type I, III, and X, which are the major components of cartilage and bone [8]. MMP13 is expressed in hypertrophic chondrocytes and osteoblasts during embryogenesis and in the adult bone and is thought to be involved in endochondral ossification and bone remodeling [9], [10]. MMP13 knockout mice show a dramatic defect on endochondral ossification [11]. It has been reported that *MMP13* is a downstream target of parathyroid hormone (PTH)-related protein [12] and the transcription factor *Runx2* in growth plate chondrocytes [13]. However, transcriptional regulation of *MMP13* expression in osteoblasts is not well understood.

**Table 1:** Effect of Osx on MMP family member expression.

Gene	Osx-WT	Osx-KO	Fold change	Transcript ID
mmp13	6735.02	108.31	−62.18	Mm.5022.1
mmp9	8281.55	542.04	−15.28	Mm.4406.1
mmp16	959.36	436.87	−2.2	Mm.42042.2
mmp8	160.22	83.00	−1.93	Mm.16415.1
mmp17	109.76	76.30	−1.44	Mm.42047.1
mmp2	6019.29	5479.28	−1.1	Mm.29564.1
mmp14	3703.01	3615.95	1	Mm.19945.1
mmp23	722.87	736.35	1	Mm.29373.1
mmp11	136.75	142.83	1	Mm.4561.1
mmp24	317.01	336.34	1	Mm.21306.2
mmp19	absent	absent		Mm.131266.1
mmp10	absent	absent		Mm.14126.1
mmp3	absent	absent		Mm.4993.1
mmp12	absent	absent		Mm.2055.1
mmp1	absent	absent		Mm.156952.1
mmp7	absent	absent		Mm.4825.1
mmp20	absent	absent		Mm.103671.1
mmp15	absent	absent		Mm.7283.1

To identify the possible downstream targets of Osx in osteoblasts, we performed quantitative real-time RT-PCR to compare RNA levels of different genes of calvarial cells between wild type and *Osx* knock-out mice. In this study, quantitative real-time RT-PCR results demonstrated that *MMP13* expression was suppressed in the absence of Osx, and enhanced when Osx was overexpressed. This suggests that Osx may control *MMP13* gene expression. Additional evidence from this study indicates that Osx targets the *MMP13* gene promoter directly. This provides a new, additional mechanism through which Osx controls osteoblast activity.

**Figure 2:**
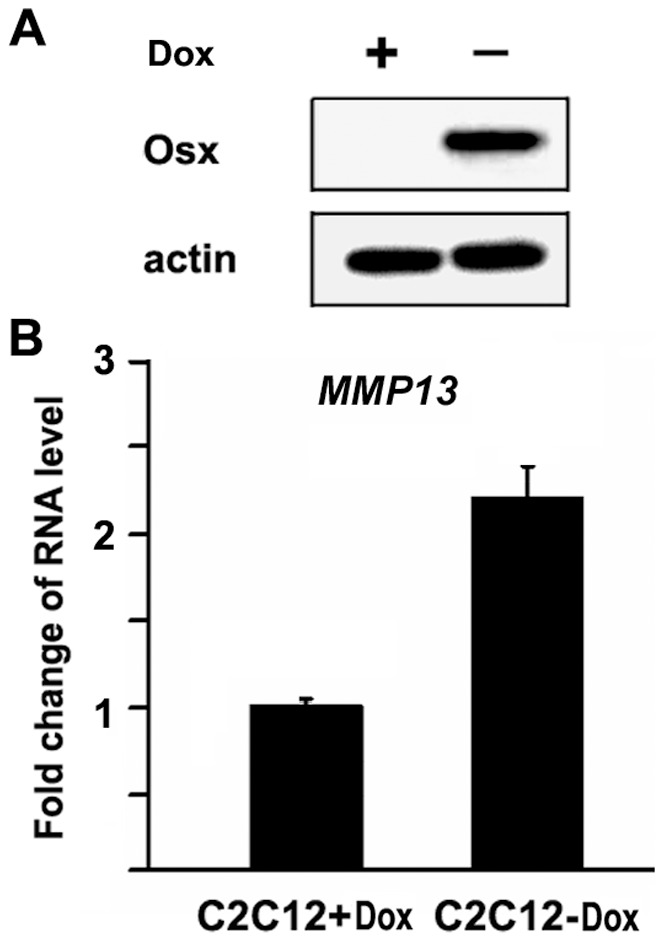
Overexpression of Osx activates MMP13 gene expression in C2C12 mesenchymal cells. (A) Western immunoblot analysis of the Dox-regulated Osx-expressing C2C12 cells. Osx expression is turned on in the absence of Dox. Beta-actin was used as a loading control. (B) MMP13 mRNA levels in a stable Tet-off C2C12 mesenchymal cell line. RNA was obtained from cultures treated with or without Doxycycline. Osx expression is induced in the absence of Doxycycline in this stable cell line. MMP13 mRNA levels were quantitated by real-time RT-PCR. The MMP13 RNA level obtained from the cells cultured with Dox was normalized to a value of 1. Values are presented as the mean ± S.D.

## Methods

### Animals and Genotyping

Wild type and *Osx*-null mice are on a C57BL genetic background. All mice were bred and maintained in a specific pathogen-free facility. Mice were genotyped using genomic DNA isolated from the tails. PCR genotyping was performed with two sets of primers: Osx5 and Osx3 for the wild-type allele and bpA and Osx3 for the mutant allele, producing 286 bp and 395 bp PCR fragments, respectively as previously described [4]. All research protocols were approved by the Institutional Animal Care and Use Committee of University of Texas Southwestern Medical Center. Animal protocol number is 2010-0008.

**Figure 3:**
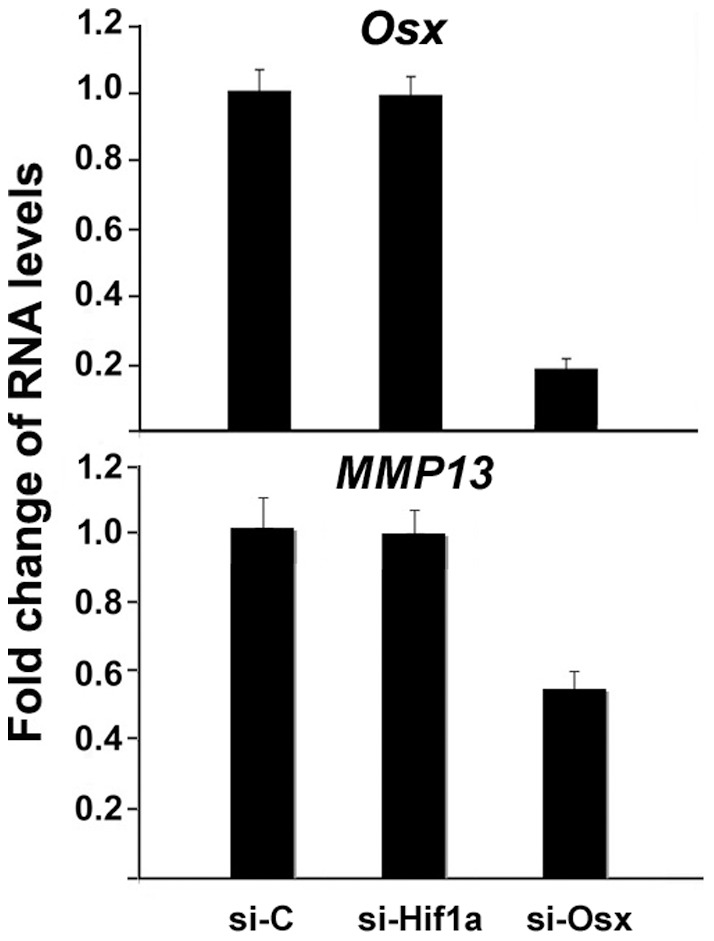
siRNA-directed knockdown of Osx impairs MMP13 gene expression in MC3T3 osteoblasts. RNA expression levels were determined by quantitative real-time RT-PCR. MC3T3 osteoblasts were transfected with siRNA targeting mouse Osx. RNA was isolated 24 hr post-transfection and quantitated by real-time RT-PCR. The RNA level from the control siRNA group was normalized to a value of 1. Values were presented as the mean ± S.D. si-C: Lamin A/C Control siRNA as a non-specific control; si-Hif1a: siRNA against Hif1a as another control; si-Osx: si-RNA against Osx.

### RNA isolation and real-time RT-PCR

Total RNA was purified from the calvaria of E18.5 wild type and *Osx*-null mouse embryos with TRIzol reagent (Invitrogen) with additional purification using an RNeasy mini kit (Qiagen). Total RNA from C2C12 cells was isolated directly using an RNeasy Mini Kit according to the manufacturer's protocols (Qiagen). RNA was subjected to quantitative real-time RT-PCR, using the TaqMan One-Step RT-PCR Master Mix reagent (Applied Biosystems). Relative transcript levels were analyzed by real-time PCR in a 20 μl reaction volume on 96-well plates, using an ABI 7500 real-time PCR system (Applied Biosystem). Transcript levels were normalized to glyceraldehyde-3-phosphate dehydrogenase (GAPDH) levels. All reactions were done in duplicate and all experiments were repeated at least three times. The relative mRNA expression levels were calculated according to the comparative C_T_ (ΔΔC_T_) method as described by the manufacturer (User Bulletin #2, Applied Biosystems). Target quantity is normalized to endogenous control and relative to a calibrator, and is calculated using formula: Target amount  = 2^−ΔΔC^_T_.

**Figure 4:**
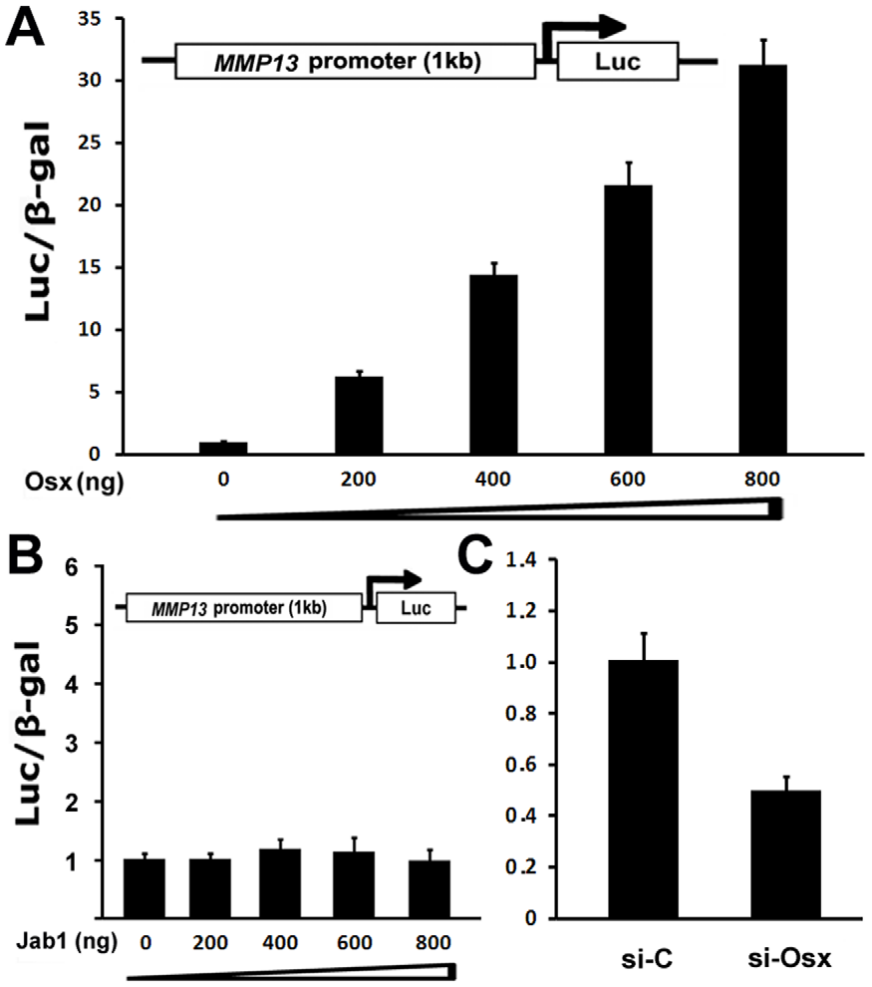
Effect of Osx on *MMP13* promoter activity. (**A**) Osx activates the *MMP13* promoter in a dose-dependent manner. HEK293 cells were transfected with a 1 kb *MMP13* promoter-luciferase reporter gene without or with increasing amounts of an Osx-expression plasmid as indicated. Luciferase activity was normalized by β-galactosidase activity. Values are presented as the mean ± S.D. (B) Jab1 does not activate *MMP13* promoter activity. HEK293 cells were transfected with a 1 kb *MMP13* promoter-luciferase reporter gene without or with increasing amounts of a Jab1-expression plasmid as indicated. Luciferase activity was normalized by β-galactosidase activity. Values are presented as the mean ± S.D. (C) MMP13 promoter activity is inhibited in the presence of Osx siRNA. MC3T3 osteoblastic cells were transfected with a 1 kb *MMP13* promoter-luciferase reporter gene with siRNA Control or Osx siRNA as indicated. Luciferase activity was normalized by β-galactosidase activity. Values are presented as the mean ± S.D.

### Plasmid constructs and subcloning

Subcloning was performed as previously described with modifications [14]. Progressive deletion fragments of the *MMP13* promoter region were generated by PCR using mouse genomic DNA as a template and subcloned into the XhoI and MluI sites of the pGL-3 vector. Primers were obtained from Integrated DNA Technologies (IDT) (Coralville, IA). The primer sequences were as follows: 1) MMP13-Xho-3 5′GCG CCT CGA GTC TCT CCT TCC CAG GGC AAG CAT, 2) MMP13-Mlu-1K 5′ GCG CAC GCG TTG ACC ATG GGG CTA GAA AGT, 3) MMP13-Mlu-540 5′GCG CAC GCG TGT CTG TGG CAG GAC TCA ATC, 4) MMP13-Mlu-210 5′GCG CAC GCG TGC TGA GGC TGT TTA TTT TGC C, 5) MMP13-Mlu-80 5′GCG CAC GCG TGT TTA CCT TCG CCT CAC TAG. Point mutations were introduced in the MMP130-80 promoter construct using the QuickChange site-directed mutagenesis kit (Stratagene) and the following primers: 1) MMP130-80-M-1 5′ GAA GTT AAC ACA TAT TTT AAA GTG GTG ACT CAT C, 2) MMP130-80-M-2 5′ GAT GAG TCA CCA CTT TAA AAT ATG TGT TAA CTT C. All deletion and mutant constructs were verified by DNA sequencing.

**Figure 5:**
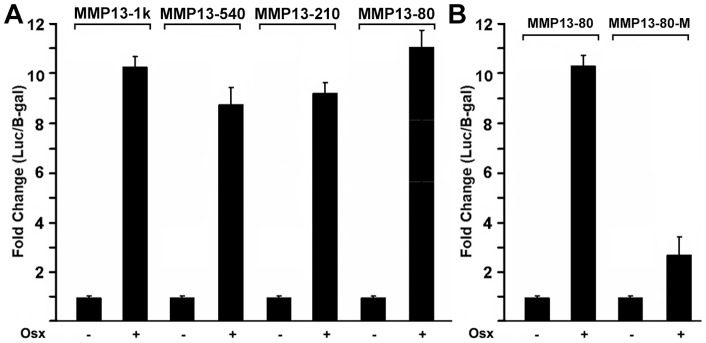
Identification of the Osx binding site in the promoter of *MMP13* gene. (A) Deletion analysis of the *MMP13* promoter-reporter constructs. MMP13-1 kb, MMP13-540 bp, MMP13-210 bp and MMP13-80 bp promoter-reporter plasmids (300 ng each) were cotransfected with 400 ng of the Osx expression plasmid in HEK293 cells. Twenty-four hours post-transfection, cell extracts were prepared and analyzed for luciferase activity and normalized to β-galactosidase activity. (B) The GC-rich element in MMP13-80 is responsible for *MMP13* promoter reporter activation by Osx. The promoter mutant MMP13-80-M was transfected into HEK293 cells and analyzed as described in panel A. Luciferase activity was normalized by β-galactosidase activity.

### Cell culture and transient transfection assay

HEK293 cells (ATCC) were cultured in Dulbecco's modified Eagle's medium (GIBCO) supplemented with 10% fetal bovine serum and 100 units/ml penicillin plus 100 μg/ml streptomycin at 95% air/5% CO_2_ humidified incubator. Cells were plated in 12-well plates, cultured to 60–80% confluence and transfected with FuGENE 6 (Roche) according to the manufacture's instruction. Cells were cotransfected with 300ng of *MMP13* promoter luciferase reporter, an Osx expression plasmid (pEX-Osx) as indicated, and 25 ng of pSV2-beta-gal. After transfection, cells were incubated for 24 h before harvest. The reporter assays were analyzed with a BD Monolight system (BD Biosciences). Luciferase activity was normalized to β-galactosidase activity. All transfection experiments were repeated at least three times. Values were presented as the mean ± standard deviation (S.D.). Stable C2C12 mesenchymal cells expressing Osx were generated with the pTet-off Advanced Inducible Gene Expression System (Clontech) as previously described [6]. C2C12 cells were cultured in Dulbecco's modified Eagle's medium with the following additives to maintain selection and control Osx expression; G418 (200 µg/ml), hygromycine (150 µg/ml), and with or without doxycycline (Dox, 20 ng/ml). Osx expression was induced by the addition of media lacking doxycycline, a member of the tetracycline group of antibiotics.

**Figure 6:**
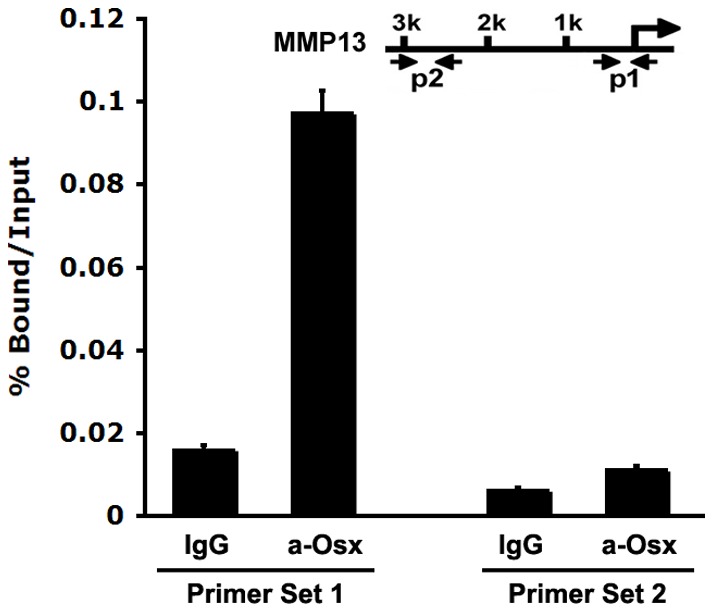
Endogenous Osx in primary osteoblasts is associated with the native *MMP13* promoter in vivo. Chromatin Immunoprecipitation (ChIP) assays were conducted using primary calvarial osteoblasts isolated from new born wild-type mice. Anti-Osx antibody (a-Osx) was used for ChIP analysis, and IgG was used as a negative control. The precipitated chromatin was analyzed by quantitative real-time PCR. As described in the Methods, primer Set 1 corresponds to a segment covering the GC-rich element within 80 bp *MMP13* promoter. As a negative control, Primer Set 2 covers a distal 3 kb region of the *MMP13* promoter, which does not contain GC-rich sequences.

**Figure 7:**
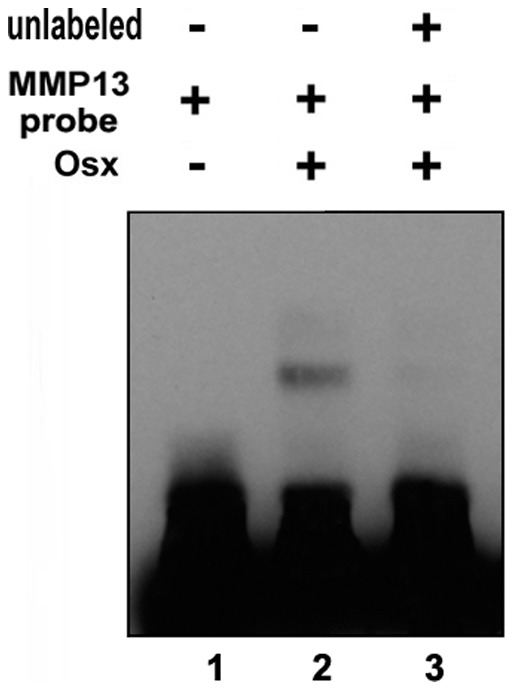
Osx binds to MMP13 promoter oligos in Gel Shift Assay. DNA oligonucleotides of MMP13 were labeled by Biotin. Osx protein and biotin-labeled DNA probe were incubated. Protein-DNA complexes were separated on 4% polyacrylamide gels, and visualized by a Chemiluminescent Nucleic Acid detection Module. Two hundred-fold molar excess of unlabeled MMP13 promoter oligos (lane 3) were used. Baculovirus-expressed Osx was used as the protein resource.

### siRNA interference

MC3T3 cells were transfected by siRNA against mouse Osx with Lipofectamine 2000. siRNA oligos were purchased from Thermo Scientific Dharmacon, and siGENOME Lamin A/C Control siRNA as well as siRNA against Hif1-α were used as non-specific controls. Cells were cultured in 6-well plates. One day before transfection, cells were plated in 1ml of the growth medium without antibiotics. Cells were 30–50% confluent at the time of transfection. For each sample, siRNA:Lipofectamine. 2000 transfection complex was prepared as follows: (1) Dilute 2 μl of 50 μM siRNA in 50 μl of Opti-MEM I Reduced Serum Medium without serum; (2) Mix Lipofectamine. 2000 gently, then dilute 3 μl in 50 μl of Opti-MEM I Medium; (3) Combine the diluted siRNA with the diluted Lipofectamine. 2000; (4) Add 100 μl of siRNA:Lipofectamine. 2000 complex to each well. After 4 hr incubation, the growth medium was replaced. Cells were cultured at 37°C in a CO_2_ incubator for 24 hr before harvest.

### Protein purification and Western blot

Protein was isolated by acetone precipitation from the RNeasy cell lysates according to the manufacturer's protocol (QIAGEN). The protein pellet was dissolved in 1% SDS buffer, warmed for 15 min at 55°C, and centrifuged for 5 min at 14000 rpm. Protein concentrations in the supernatant were determined using a BSA Protein Assay Kit (Pierce). Proteins were separated on 10% SDS-PAGE gels and transferred to a PVDF membrane followed by Western blot analysis. Briefly, 3% milk in TBS containing 0.1% Tween-20 was used to block non-specific binding. The blot was subsequently incubated with an anti-Osx rabbit polyclonal antibody (1∶200, Santa Cruz Biotechnology) followed by a secondary antibody (peroxidase-conjugated anti-rabbit IgG 1∶5000, Sigma). After each antibody incubation, blots were extensively washed in TBS containing 0.1% Tween-20. For detection, the ECL kit (Amersham Life Sciences) was used according to the directions of the manufacturer.

### Gel Shift Assay

Baculovirus-expressed Osx was used as the protein resource as described [6]. The DNA sequences of the oligonucleotides used for Gel Shift Assay were as follows: MMP13 5′AGG AAG TTA ACA CAC ACC CCA AAG TGG TGA CTC ATC. DNA oligonucleotide was labeled using a Biotin 3′ end DNA Labeling Kit (Cat#: 89818, Pierce Biotechnology Inc.). Osx protein and biotin-labeled DNA probe were incubated in 1x binding buffer for 20 min at room temperature using LightShift Chemiluminescent EMSA kit (Cat#: 20148). Protein–DNA complexes were separated on 4% polyacrylamide gels in 0.5x TBE buffer, and transferred onto Biodyne B Nylon Membrane (Cat#: 77016). The membrane was blocked in 1x blocking buffer, washed five times with 1x wash buffer, and visualized by a Chemiluminescent Nucleic Acid detection Module (Cat#: 89880). Two hundred-fold molar excess of unlabeled MMP13 promoter oligos was used as competitor DNA.

### Chromatin immunoprecipitation (ChIP) assay

The Chromatin Immunoprecipitation (ChIP) Assay Kit was from Millipore. ChIP assays were performed as previously described [15] with some modifications. Briefly, calvarial cells were isolated from wild-type newborn mice, and were cultured in DMEM supplemented with 10% FBS. Formaldehyde was used to cross link the cells for 10 min, and crosslinking was quenched with glycine. Cells were harvested, rinsed with PBS, and cell pellets were resuspended in 1 ml of lysis buffer. After sonication, 100 µl of sheared chromatin was diluted to 1 ml with IP dilution buffer for each immunoprecipitation. The chromatin solution was pre-cleared with 60 µl of protein G Agarose beads at 4°C for 1 hr. The pre-cleared chromatin was collected and incubated at 4°C overnight with 5 µg of anti-Osx antibody or IgG as a negative control. The immune complexes were precipitated with 60 µl of protein G Agarose beads at 4°C for 1 hr. The antibody-protein-DNA immunocomplexes were washed and subsequently eluted twice with 100 µl of elution buffer. Formaldehyde cross-linking was reversed by heating at 65°C overnight with the addition of 5 M NaCl. All the samples were digested with RNase A and proteinase K. The DNA was purified using spin columns, and analyzed by real time PCR. The primer sets used for amplification of MMP13 promoter regions were obtained from IDT, and the sequences were as follows: Primer Set 1, MMP13-1: 5′ GTT TAC CTT CGC CTC ACT AG and MMP13-2: 5′ TCT CTC CTT CCC AGG GCA AGC AT; Primer Set 2, MMP13-D-1: 5′ CGC ATT AGA GTT GAG AAG TAG and MMP13-D-2: 5′ ACA GTG GAT AGA GTA CAC AG. Data were normalized using GAPDH.

## Results

### MMP13 expression is dramatically reduced in the absence of Osx

To identify possible downstream targets of Osx in the developing mouse skeleton, we performed microassay to compare gene expression profiles in osteoblasts between Osx wild type and Osx knockout mice as previously described [6]. RNA was isolated from calvaria of E18.5 mouse embryos. We were interested in members of MMP family. As shown in Table 1, MMP13 is in the top list, but is not an exclusive target of Osx among MMP family members. Of the MMPs, MMP13 has been considered to have an essential role in bone biology in view of its exclusive presence in the skeleton during development. Therefore, we focused on MMP13 first in our study. To confirm microarray results, we performed quantitative real-time RT-PCR on RNA from wild type and *Osx* knockout mice in order to compare RNA levels of several genes. As shown in Fig. 1, *Osx* expression was readily detected in wild-type calvaria and predictably absent in *Osx*-null calvaria. Likewise, osteocalcin (OC) expression, a marker of mature osteoblasts, was abolished in Osx-null calvaria compared to wild-type tissue. In contrast, Runx2, a gene that is upstream of Osx in the osteoblast differentiation, remained unchanged in the Osx-null calvaria. Interestingly, we observed that *MMP13* expression was dramatically suppressed by greater than 60-fold in *Osx*-null calvaria compared with that in wild-type calvaria. The significant decrease in *MMP13* RNA level in *Osx* knock-out mice suggests that *Osx* regulates *MMP13* gene expression.

### Overexpression of Osx activates MMP13 gene expression

To test whether forced expression of Osx stimulated *MMP13* gene expression, we used a stable, Osx-inducible C2C12 mesenchymal cell line that was described previously [6]. In this line, protein expression of Osx is induced upon removal of doxycycline (Dox) (Fig. 2A). Total RNA was purified from this line following culture in the presence or absence of Dox and *MMP13* expression was quantitated by real-time RT-PCR. As shown in Fig. 2B, in the absence of Dox (i.e., overexpression of Osx), *MMP13* expression was enhanced 2.2 fold. These data indicate that increased expression of Osx results in increased expression of MMP13 in the C2C12 cell system.

### Inhibition of Osx by siRNA reduces MMP13 gene expression in osteoblasts

To establish a direct effect of Osx on MMP13 expression, we used siRNA to knockdown Osx expression in MC3T3 osteoblast cells. MC3T3 cells were chosen for this approach because they express readily detected levels of Osx. Real-time RT-PCR was performed to analyze gene expression levels. As shown in Fig. 3, when Osx RNA expression was decreased by 80% using siRNA targeted against Osx, MMP13 RNA levels were reduced by approximately 46%. We used Lamin A/C Control siRNA as a non-specific control to show the specificity in Fig. 3. To confirm the specificity, we also used siRNA-Hif1a, and there was no effect of siRNA-Hif1a on MMP13 expression. Therefore, these data support a role for Osx in enhancing MMP13 gene expression in osteoblasts.

### Osx activates the MMP13 promoter activity in a dose-dependent manner

Results from primary mouse calvarial cells, the stable C2C12 mesenchymal cell line and the MC3T3 osteoblastic cell line strongly indicate that Osx upregulates MMP13 expression. To test the effect of Osx on the MMP13 promoter activity, we did subcloning to generate a luciferase reporter construct driven by 1 kb *MMP13* native promoter. HEK293 cells were transiently transfected with the MMP13 promoter-luciferase reporter and Osx expression vector. HEK293 cells were chosen because they are very easy to grow and have high transfection efficiency for transient transfection assay used in our current study. As shown in Fig. 4A, increasing amounts of Osx induced markedly higher MMP13 promoter activities, and transfection with 800 ng Osx resulted in a 31-fold increase of MMP13 promoter activity, demonstrating that Osx activates MMP13 promoter activity in a dose-dependent manner. We used non-specific expression vector Jab1 as a control, provided by Dr. Guang Zhou from Case Western Reserve University. There was no effect of Jab1 on MMP13 reporter expression as shown in Fig. 4B. We further performed transfection experiments in MC3T3 cells in the presence or absence of Osx siRNA. Lamin A/C Control siRNA was used as a non-specific control. As shown in Fig. 4C, MMP13 promoter activity was inhibited by 48% in the presence of Osx siRNA, compared with control siRNA. These results are in agreement with our data from real time RT-PCR experiments in a C2C12 stable cell line (Fig. 2), indicating that Osx regulates MMP13 positively.

### Identification of the Osx binding site in the promoter of MMP13 gene

To define the precise Osx regulatory region in the *MMP13* promoter, we generated a series deletion constructs of the *MMP13* promoter reporter and tested them in the transfection assay. As shown in Fig. 5A, Osx activated all of the *MMP13* promoter reporters similarly, including the MMP13-1 kb, MMP13-540 bp, MMP13-210 bp and MMP13-80 bp constructs. These data indicate that the putative regulatory or binding elements for Osx reside within the MMP13 80 bp promoter region. Previous studies show that Osx belongs to the Sp/XKLF family of transcription factors that bind to GC-rich sequences of target gene promoters to regulate gene expression [4]. Sequence analysis of the active 80 bp region of *MMP13* promoter reveal one potential GC-rich binding site for Osx located at the proximal region close to the transcriptional start site. To study whether the potential binding site is responsible for *MMP13* promoter activation by Osx, we disrupted the element by site-directed mutagenesis. The mutant was designated MMP13-80-M in which the cytosines (Cs) in the element were replaced with the thymines (Ts). As shown in Fig. 5B, MMP13-80-M mutant almost abolished the MMP13 reporter activation by Osx in transient transfection assay. Cumulatively, these data revealed that the GC-rich sequence functions as an Osx-responsive element that mediates the activation of the MMP13 promoter by Osx.

### Endogenous Osx associates with the native MMP13 promoter in vivo

The studies above indicate that Osx can positively regulate MMP13 expression and activate the *MMP13* promoter *in vitro* through the GC-rich sequence in *MMP13* promoter. However, it is currently unknown whether endogenous Osx associates with the native *MMP13* promoter *in vivo*. To address this question, chromatin immunoprecipitation assays were carried out to examine whether Osx could bind to native *MMP13* promoter. Primary calvarial osteoblasts were isolated from new born wild-type mice for ChIP assay. Crosslinked extracts were immunoprecipitated with antibodies against Osx or control IgG. Following reversal of the crosslinks, DNA was recovered and analyzed by quantitative real time PCR using primers designed to amplify the Osx-responsive region covering the GC-rich sequence of 80 bp promoter of MMP13 gene (Primer Set 1) or a distal upstream 3 kb, non-responsive region (Primer Set 2) as a control to demonstrate response element selectivity. Fig. 6 demonstrated that Osx was associated with the *MMP13* promoter region containing the the GC-rich sequence (Primer Set 1) compared with IgG control group. However, Osx was not associated with the *MMP13* distal 3 kb promoter region lacking a GC-rich sequence (Primer Set 2), demonstrating that the observed Osx-DNA association was specific. Thus, these data indicate that endogenous Osx associates with the native *MMP13* promoter in primary osteoblasts *in vivo*. To confirm Osx binding to the MMP13 promoter, we performed gel shift assay. As shown in Fig. 7, Osx was able to bind to MMP13 promoter oligos (lane 2), and Osx binding was abolished by excess unlabeled MMP13 promoter oligos (lane 3), which was used to test the binding specificity. The data indicated that Osx bound to MMP13 promoter oligos specifically.

## Discussion

Osx is an osteoblast-specific transcription factor that required for osteoblast differentiation and function. Despite the discovery of its significance in skeletal physiology a decade ago [4], relatively little is known about direct target genes for Osx and molecular mechanisms through which Osx regulates transcription. The findings in this study add new insights into these two general, poorly understood areas of Osx biology.

First, we identified MMP13 as an Osx target gene in osteoblasts. This is supported by the Osx-directed gene expression studies showing coordinate expression of Osx and MMP13 in several *in vivo* and *in vitro* model systems. For example, Osx-null calvaria displayed defective *in vivo* expression of MMP13 compared to Osx wild-type calvaria in mice. Primary osteoblast cell line MC3T3 cultures obtained from wild-type calvaria also had markedly impaired MMP13 gene expression when Osx expression was knocked-down using siRNA targeting strategies (Fig. 3) and a Tet-off inducible cell system revealed that ectopic expression of Osx resulted in an increase in MMP13 transcript level (Fig. 2B). Importantly, a direct regulation of MMP13 gene transcription by Osx was evident in the ability of recombinant Osx to activate MMP13 promoter-reporter constructs, thus indicating that the RNA expression studies were likely due to the effects of Osx expression on MMP13 gene transcription. The broader implication of these studies is that the MMP13 gene can now be added to a small, but growing list of osteoblastogenic factors and pathways that are regulated by Osx in the osteoblast [6], [16], [17], [18], [19].

In terms of mechanism, Osx is an SP/KLF family member that presumably functions by binding directly to DNA promoter elements via an SP1-like DNA-binding domain consisting of three C2H2-type zinc fingers located within its C-terminus [4]. In this study, the results of the promoter sequence mutants help to support that Osx shares with the SP/KLF family to bind to GC-rich sequence of target gene promoter. We previously reported on similar elements controlling Osx-activation of the DKK1, sclerostin and VEGF promoters [6], [16], [18]. Indeed, such GC-rich region exists in the most proximal regions of the murine MMP13 promoter residing immediately adjacent to its transcriptional start site. Importantly, our studies define the element as critical for mediating the transcriptional activation of the MMP13 promoter by Osx. Mutation of the element (MMP13-80-M) nearly abolished Osx-directed activation of the MMP13 promoter (Fig. 5B). Of course, limitations of this particular approach include the heterologous expression and somewhat artificial nature of the plasmid DNA constructs used as a measure of a biologically relevant transcriptional event. Thus, the chromatin immunoprecipitation approaches (Fig. 6) strongly support the reporter gene expression data. These studies clearly demonstrated that endogenous Osx was associated with the GC-rich proximal region of the native MMP13 promoter in primary osteoblastic cells. Gel Shift Assay showed that Osx bound to *MMP13*. These studies help establish Osx mechanisms involving direct binding of Osx to sequence specific, GC-rich promoter element to activate the expression of genes in osteoblastic cells.

Osteoblasts undergo a progressive differentiation from mesenchymal stem cells to preosteoblasts to mature osteoblasts and finally to osteocytes, with each stage being characterized by different phenotypes and expression of specific osteogenic transcription factors and makers. MMP13 is an important marker for mature osteoblasts and hypertrophic chondrocytes during endochondral ossification [10]. MMP13 levels increase continuously during osteoblastic differentiation [20], [21]. MMP13 knockout mice fail to undergo normal ossification with a delay in ossification at the primary centers [11]. Because of these findings MMP13 is considered important in bone formation and remodeling. However, despite the intricate role of MMP13 during osteoblast differentiation, regulation of MMP13 in osteoblasts is not well elucidated. Osx knockout mice are lethal, and lack bone formation completely though cartilage is normal [4]. Inactivation of Osx after birth using conditional knockout approach also causes an arrest of osteoblast differentiation and of new bone formation [22]. Interestingly, MMP13 expression in the long bones and calvaria of the Osx conditional null mutants was significantly decreased. However, it remains unclear whether Osx can control MMP13 directly. We show here that MMP13 is a direct target of Osx in osteoblasts. MMPs have been associated with cancer cell invasion and metastasis, including MMP13 [23]. Our current studies indicate that Osx controls MMP13 expression in osteoblasts, suggesting a possible involvement of Osx in bone cancer in addition to normal bone development.

Osx is considered as a master regulator of osteoblast differentiation [4], [6], [22]. Unfortunately, the cellular and molecular mechanisms governing Osx control of osteoblast differentiation and function are still not well characterized. Osx is known to function downstream of Runx2 during osteoblast differentiation [4], [24] and Osx is required for the expression of osteoblast-specific markers, such as type I collagen, osteocalcin, bone sialoprotein, osteonectin and osteopontin [4]. Thus, while additional studies need to address other cell systems and the ultimate effects of Osx and MMP13 regulation on the full osteoblastic cell differentiation pathway progressing to the stage of mineralized matrix production, these early stage studies provide a sound biological context and relevance for Osx activation of MMP13 gene expression in this model system. In this study, we mainly focus on transcriptional regulation mechanisms whereby Osx modulates MMP13 gene transcription. It will be interesting to explore in the future whether MMP13 overexpression could rescue Osx null defective bone phenotype.
