# Correction: Matrix Metalloproteinase 13 (MMP13) Is a Direct Target of Osteoblast-Specific Transcription Factor Osterix (Osx) in Osteoblasts

**DOI:** 10.1371/annotation/a5ac9734-faa6-41d3-8ba2-6d0894f62db5

**Published:** 2013-08-06

**Authors:** Chi Zhang, Wanjin Tang, Yang Li

The authors would like to acknowledge that there is overlap between some of the controls reported in the article with those included in our previous publication below (reference 17 in this article):

Zhang C, Tang W, Li Y, Yang F, Dowd DR, et al. (2011) Osteoblast-specific transcription factor Osterix increases vitamin D receptor gene expression in osteoblasts. PLoS One 6: e26504

In particular, the left panels in Figure 1 overlap with the left panels in Figure 1 reported in our PLOS ONE article above and Figure 2A in the article reports a blot already included in the previous PLOS ONE article. This overlap should have been declared in the article and the authors apologize for this oversight.

The purpose of Figure 1 in the current article is to show that Osx ablation reduces MMP13 gene expression in Osx-null mice at E18.5. The left panels of the figure report the results for Osx and osteocalcin (OC). Osx was used to confirm the Osx-null phenotype. We included multiple controls for the Osx-null phenotype in order to show that the osteoblast marker gene OC was also abolished in Osx-null calvaria.

Figure 2A in the current article was reported as a control to confirm that Osx expression is induced in the stable Osx-inducible C2C12 cell line established previously in our previous PLOS ONE article (pone.0026504). 

